# Spatial-Temporal Dynamics of Hepatitis E Virus Infection in Foxes (*Vulpes vulpes*) in Federal State of Brandenburg, Germany, 1993–2012

**DOI:** 10.3389/fmicb.2020.00115

**Published:** 2020-01-31

**Authors:** Martin Eiden, Lisa Dähnert, Susanne Spoerel, Ariel Vina-Rodriguez, Ronald Schröder, Franz J. Conraths, Martin H. Groschup

**Affiliations:** ^1^Institute for Novel and Emerging Infectious Diseases, Friedrich-Loeffler-Institut, Greifswald-Insel Riems, Germany; ^2^Tierarztpraxis Dr. Kindler, Wiesbaden, Germany; ^3^Institute of Epidemiology, Friedrich-Loeffler-Institut, Greifswald-Insel Riems, Germany

**Keywords:** hepatitis E virus, foxes, infection, transudates, *Orthohepevirus C*

## Abstract

Hepatitis E virus (HEV) is the main course for acute hepatitis in humans throughout the world. Human associated genotypes 1 and 2 as well as zoonotic genotypes 3 and 4 are grouped in the species *Orthohepevirus A*. In addition, a large variety of HEV-related viruses has been found in vertebrates including carnivores, rats, bats, and chickens, which were classified in species *Orthohepevirus B-D*. In 2015, partial genome sequences of a novel hepevirus were detected in feces of red foxes (*Vulpes vulpes*). However, no further information about virus circulation and the prevalence in foxes was available. We therefore assayed a unique panel of 880 transudates, which was collected from red foxes over 19 years (1993–2012) in Brandenburg, Germany, for HEV-related viral RNA and antibodies. Our results demonstrate a high antibody prevalence of HEV in red foxes, which oscillated annually between 40 and 100%. Molecular screening of the transudates revealed only a single RNA-positive sample, which was assigned to the carnivore species *Orthohepevirus C* based on the amplified partial sequence. These data indicate that the virus is circulating widely in the fox population and that foxes are carriers of this virus.

## Introduction

HEV is a member of the genus *Orthohepevirus* of the *Hepeviridae* family and causes acute liver diseases in humans. The virus consists of a single stranded RNA genome of positive polarity with a length of approximately 7.2 kb, which contains three open reading frames (ORF1-3). ORF1 encodes a nonstructural and further processed protein, which includes the RNA-dependent RNA polymerase (RdRp), ORF2 encodes the viral capsid protein and ORF3 for a small phosphoprotein, which is necessary for viral release (LeDesma et al., 2019).

The human associated as well as zoonotic genotypes are grouped into the *species Orthohepevirus A*, which includes a total of 8 genotypes, originating from pig, wild boar, rabbit, and camel species. *Orthohepevirus B* consists of avian hepatitis E virus species causing the “splenomegaly syndrome” as well as the “big liver and spleen disease” in poultry, whereas *Orthohepevirus C* viruses were isolated from rodents (rats voles and shrew) and carnivores (such as ferrets, mink and foxes). HEV from bats are classed in the species *Orthohepevirus D*. Finally, fish-related HEV belongs to genus *Piscihepevirus* (Spahr et al., 2018).

To determine and quantify the circulation of HEV in the animal population, numerous monitoring studies have been performed in Europe in the past. Based on serological and molecular results, pigs and wild boars are considered as the main reservoirs of the virus and a potential source of zoonotic transmissions (Van der Poel, 2014). In Germany, activities mainly focused on zoonotic genotype 3 (species *Orthohepevirus A*) found in pig, wild boar, deer, and wild rabbits. The resulting seroprevalences ranged from 1% in deer (Neumann et al., 2016), 35–37% in rabbits (Hammerschmidt et al., 2017; Ryll et al., 2018), 41% in wild boar (Schielke et al., 2009) up to 100% in some pig holdings (Dremsek et al., 2013). Molecular studies revealed that especially rabbits, wild boar, and pigs frequently carried or excreted HEV (Kaci et al., 2008; Baechlein et al., 2013; Vina-Rodriguez et al., 2015; Anheyer-Behmenburg et al., 2017; Hammerschmidt et al., 2017; Ryll et al., 2018).

Epidemiological studies in other species including rats, voles, and small carnivores (e.g. mink and ferrets) assigned HEV sequences to the *Orthohepevirus C* group (Raj et al., 2012; Krog et al., 2013; Ryll et al., 2017, 2019). In Norway rats from Germany, the seroprevalence varied between 14.7 and 41.2% (Johne et al., 2012), while wild carnivores like raccoons and raccoon dogs were seropositive in the range of 37 to 54% (Dähnert et al., 2018). So far, only one red fox (*Vulpes vulpes*) from the Netherlands carried HEV-derived RNA in feces (Bodewes et al., 2013) and sequencing suggested that the virus clustered to the *Orthohepevirus C* group. However, no further serological or molecular data were available for foxes, although this species is considered to be a potential virus reservoir. We therefore undertook a comprehensive HEV surveillance study with a unique panel of fox transudate samples, which were collected over 20 years (1993–2012) in the federal state of Brandenburg, Germany.

## Materials and Methods

### Sample Material

Fox cavity transudate samples were collected during an *Echinococcus multilocularis* surveillance program conducted in the German federal state of Brandenburg. Data on the hunting date, sex, age, and location were recorded for all samples (Conraths et al., 2003).

### Hunting Statistics

The population density of red foxes in Brandenburg was deduced by the number of the yearly hunted foxes (Supplementary Figure S1) using the hunting index of population density (HIPD), which is calculated from number of foxes shot per km^2^ and per year (Bögel et al., 1974).

### Serology

Transudates were analyzed by the species-independent HEV-Ab ELISA (AXIOM, Buerstadt, Germany) according to the manufacturers protocol. This commercial kit is a double-antigen sandwich ELISA, which is based on recombinant HEV ORF2 protein as antigen. It is demonstrated as a species independent assay and can detect all immunoglobulin classes (IgG, IgM, and IgA). The specificity of the assay was checked by a modified strip immunoassay recomLine HEV IgG/IgM (Mikrogen GmbH, Neuried, Germany) as well as an in-house western blot. The strip immunoassay was carried out according to the manufacturers manual, but the secondary human antibody was replaced by horseradish peroxidase (HRP) conjugated goat anti-dog IgG (Dianova GmbH, Hamburg, Germany). Positive samples exhibit significant HEV specific signal especially for C-terminal part of ORF-2 capsid protein (Supplementary Figure S2). Western blotting was performed with recombinant ORF-2 protein encompassing 239 amino acids of the HEV genotype 3 capsid protein. In short, after SDS gel electrophoresis in a 16% acrylamide gel, protein was transferred to a PVDF membrane by semi-dry electroblotting. After blocking 30 min at room temperature with 5% skim milk (Difco) in PBS/0.1% Tween 20, the membranes were incubated for 1 h at RT with corresponding fox transudates in 1:100 dilution. The membranes were then washed three times for 10 min in PBS/0.1% Tween 20, and a secondary HRP-conjugated goat anti-dog antibody (Dianova GmbH, Hamburg, Germany) diluted 1:1,000 was incubated for 1 h. After a second washing step, the proteins were visualized by chemiluminescence detection with ECL substrate (ThermoFisher) and VersaDoc Imaging system (Supplementary Figure S3).

### RNA Isolation and Molecular Analysis

RNA was extracted by QIAmp Viral Mini Kit (Qiagen GmbH, Hilden, Germany) as instructed by the manufacturer and HEV-specific genome copies amplified by a nested real-time RT-PCR protocol using primers targeting the RNA-dependent RNA polymerase (RdRp) region (Vina-Rodriguez et al., 2015; Hammerschmidt et al., 2017). In brief, reverse transcription was carried out with Superscript® III Reverse Transcriptase (Thermo Fisher Scientific Inc., USA) and primers HEV.RdRp_F1 (5′-TCGCGCATCACMTTYTTCCARAA-3′) and HEV.RdRp_R1 (5′-GCCATGTTCCAGACDGTRTT CCA-3′) according to the manufacturers’ protocol, followed by 40 cycles of 20 s denaturation at 95°C, 30 s annealing at 50°C, and 1 min elongation at 72°C, finishing with 7 min at 72°C. Subsequently, a nested PCR followed using Maxima SYBR Green/Fluorescein qPCR Master Mix Kit (Thermo Fisher Scientific Inc., USA) and primers HEV.RdRp_F2b (5′-GTGCTCTGTTTGGCCCNTGG TTYMG-3′) and HEV.RdRp_R2 (5′-CCAGGCTCACCR GARTGYTTCTTCCA-3′) according to an established protocol (denaturation for 10 min at 95°C and 40 cycles of 15 s denaturation at 95°C, 30 s annealing at 50°C, 30 s elongation at 95°C, 50°C and 30 s elongation at 95°C). Finally, a melting curve analysis was performed starting with a temperature gradient from 68 to 94°C in increments of 0.2°C. Positive samples were determined by melting peaks and amplicons were excised and subsequently sequenced (Eurofins Genomics, Germany). Standard precautions were taken to prevent PCR contamination including a closed system for PCR amplification and detection. In addition, preparation of PCR mastermix and primers, RNA-extraction, and final addition of RNA were carried out in separate laboratories.

### Phylogenetic Analysis

Phylogenetic analysis was carried out with Geneious Tree Builder using Neighbor-Joining analysis. Genetic distances were calculated using the Tamura-Nei Method. Bootstrap values >70 are displayed at nodes. Phylogenetic analysis was carried out with a 280-nt fragment of the RNA-dependent RNA polymerase gene. Sequence of avian Hepatitis E virus was used as outgroup to root the tree.

### Statistical Methods

A 95% confidence intervals (CI) were calculated using R (R Development Core Team, 2008) in *R studio*. Differences in prevalences were compared by the Fisher exact test.

## Results

**Figure 1:**
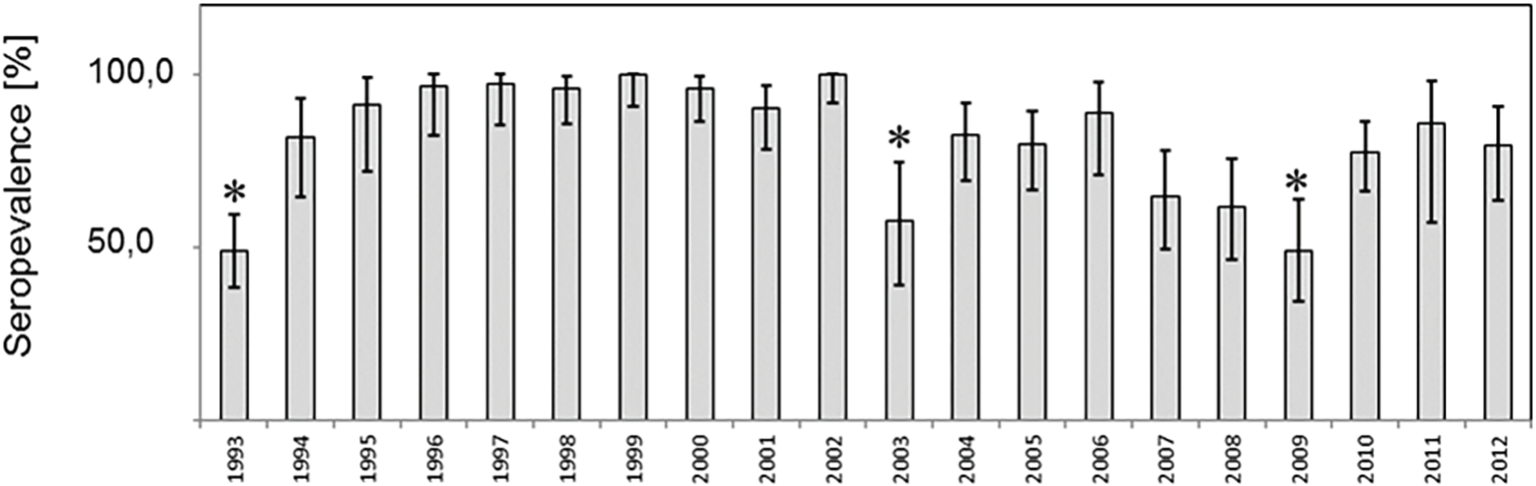
Prevalence of antibodies to Hepatitis E virus among red foxes in the federal state of Brandenburg, Germany. **(A)** Prevalence estimates per year and the respective two-sided 95% confidence intervals are shown. Differences in prevalences were compared by the Fisher exact test und significant differences (*p* < 0.05) indicated by asterics.

**Figure 2:**
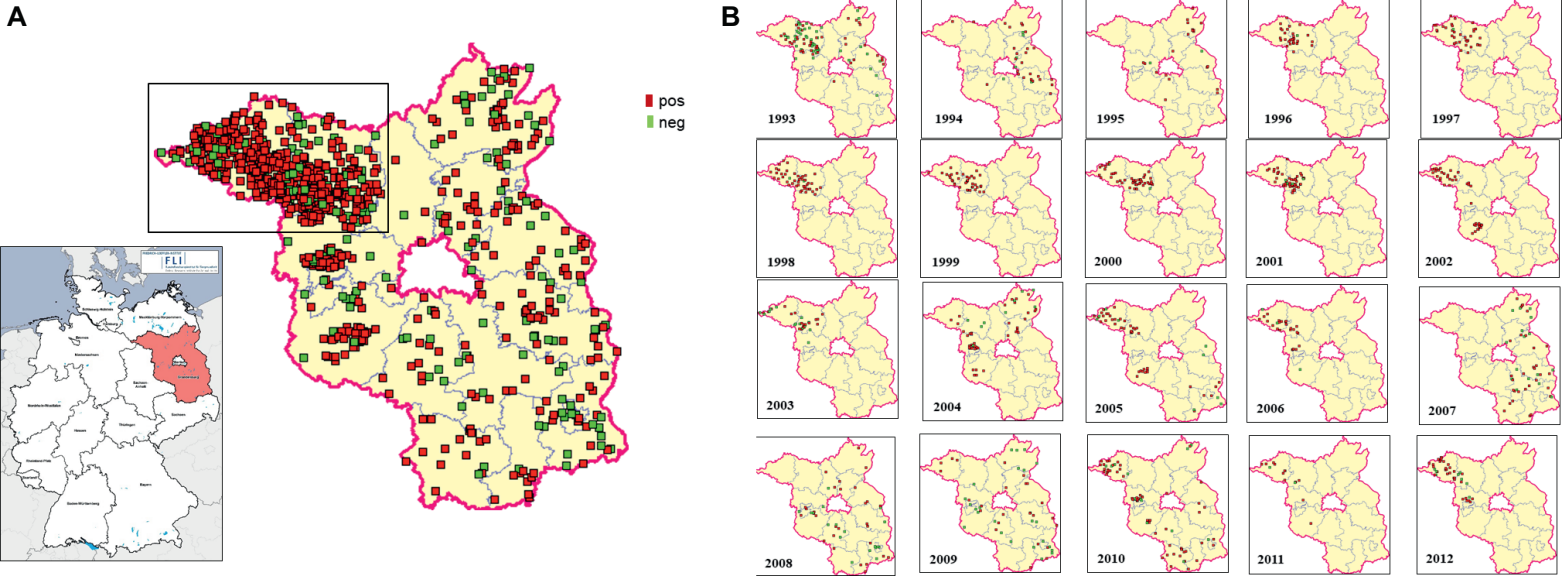
Spatial distribution of HEV-tested fox samples in the federal state of Brandenburg, Germany. The geographic origin of each examined fox sample is plotted on the map. The districts of Ostprignitz-Ruppin and Prignitz, from where the majority of samples were obtained, are marked. Positive samples are represented by red dots, negative samples by green dots. Overview comprising 20 years **(A)** and annual distributions **(B)**.

Eight hundred and eighty fox transudates collected from 1993 to 2012 in the federal state of Brandenburg, Germany, were analyzed using in Axiom ELISA and displayed an overall high seroprevalence of about 81% on average, which varied between 48.9 and 100% over the years (Figure 1). The exact number is depicted in Supplementary Table S1. In order to confirm the immunoreactivity against HEV independently, selected samples were tested by a strip immunoassay (Supplementary Figure S2) and an in-house western blot (Supplementary Figure S3). The spatial distribution of positive and negative samples throughout the district are displayed in an overview map (Figure 2A) and in annual maps (Figure 2B). Samples were collected from all 12 districts within the federal state of Brandenburg, but the majority of the 516 samples originated from the two districts Ostprignitz-Ruppin and Prignitz, which are located in the North-West of Brandenburg (Figure 2A, selected section).

About 52.0% of the samples were from male individuals and 32.8% from female individuals. The majority of the samples (74%) derived from adult animals (born in a previous year) and 9.2% from juvenile foxes, i.e., animals born in the hunting year (1 April to 31 March) when they were sampled (Table 1). No statistically significant age- or sex-associated differences in the seroprevalence were found (Table 1).

**Table 1:** Overview of analyzed fox samples. The age of each fox was determined as adult (born in a previous year) or juvenile (born in the year of sampling).

Sample characteristics		Positive [%]	Negative [%]	Total
Sex	Male	367 [80,1]	91 [19,9]	458
	Female	236 [81,7]	53 [18,3]	289
	Unknown	86 [64,7]	47 [35,3]	133
Age	Adult	522 [80,1]	130 [19,9]	652
	Juvenile	70 [86,4]	11 [13,6]	81
	Unknown	97 [66,0]	50 [34,0]	147

From 1993 to 1994, a significant increase from 48.9 to 81.1% was observed and after 10 years, a significant decline from 100 to 57.6%, followed by a significant rise from 49 to 77% 6 years later 2009. A similar finding in the seroprevalence was observed for the samples collected in 1993 and 2003 when looking only to the districts Ostprignitz-Ruppin and Prignitz (Supplementary Figure S4).

**Figure 3:**
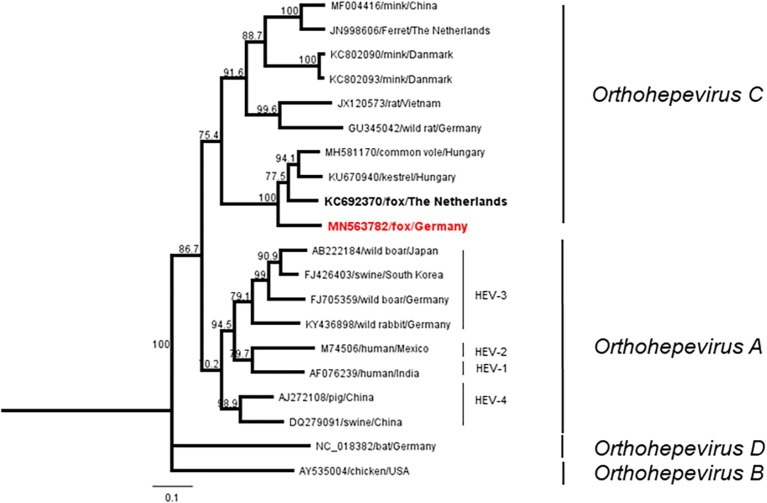
Neighbor-joining phylogenetic tree based on partial RdRp sequences. Red boldface indicates fox sequence from Germany (this study). Boldface displays fox sequence from the Netherlands. Scale bar indicates mean number of substitutions per site.

All samples were subjected to a broad-range nested RT-PCR targeting the RNA-dependent RNA-polymerase (RdRp) gene. From one sample, a partial sequence of 280 nucleotides could be recovered (accession number: MN563782), which clustered to the *Orthohepevirus C* group. Phylogenetic analysis revealed high identity of 82–83% to a HEV isolate from a fox from the Netherlands and to a sequence of kestrel in Hungary (Figure 3). Pairwise comparison of the corresponding 90 amino acid fragment showed sequence identity of 90 and 89%, respectively, corresponding to 9 and 12 amino acid changes (Supplementary Figure S5).

## Discussion

In this study, we investigated nearly 900 transudate samples from foxes collected in the federal state of Brandenburg collected during a period of 20 years and found a high seroprevalence for HEV in this population. This first report on HEV antibodies in foxes indicates therefore frequent HEV infections in this wild carnivore species. Neither age nor sex effects were observed which speaks for a general infection *via* the oral-fecal route probably by exposure to infectious feces.

Time kinetics showed an oscillation between 40 and 100% per year and a declining prevalence in 1993, 2003, and 2009. The underlying reasons are unknown, but it seems possible that the changes may have been triggered by variation of environmental factors or changes in the social organization pattern. In general, adult foxes are solitary hunters with low contact rates, but individuals have short- and long-term relationships and a seasonal community structure, e.g., during to cooperative raising of cubs (Dorning and Harris, 2019).

Seroprevalence studies from other members of the superfamily *Canidae* are limited. Raccoons and raccoon dogs had a prevalence of about 53.8 and 34.3%, respectively (Dähnert et al., 2018). A disparate picture is seen in dogs where seroprevalences ranged from 0.8% in the UK to 56.6% in Germany up to 88.5% in China (Liu et al., 2009; McElroy et al., 2015; Dähnert et al., 2018).

Only a single HEV genome sequence was extracted, which can be attributed to the age of the samples and a probably short and transient viraemic phase in foxes. In general, transudate fluids and blood/serum samples contain significantly smaller amounts of viral RNA compared to feces or tissue material. The obtained sequence showed a high identity to a fox feces derived HEV sequence isolated in The Netherlands (Bodewes et al., 2013), and both constitute a potential subclade together with other species including voles and a kestrel. Both sequences cluster with sequences from a kestrel and from vole-associated HEV strains (Reuter et al., 2016; Kurucz et al., 2019) and constitute one distinct clade within the *Orthohepevirus C* species. This includes also sequences isolated most recently from voles in Germany (Ryll et al., 2019).

The results demonstrate endemic HEV infections in a fox population over at least 20 years. As no HEV associated clinical signs in foxes are known to date, this species my perhaps even constitute a reservoir species (Haydon et al., 2002). However, direct information about virus shedding and subsequent infection is lacking. In addition, no information about the virulence of fox HEV and any possible impact on morbidity and mortality is available so far. At least no influence on yearly population density as displayed by means of the *hunting index of population density* (HIPD) could be observed (Supplementary Figure S6).

Questions regarding zoonotic character of fox-derived HEV remain open due to the lack of further sequence information and life virus. In general, foxes are the most widespread predators throughout the world and have been recently recognized as potential reservoirs of zoonotic pathogens including trematodes, cestodes, and nematodes (Mackenstedt et al., 2015) as well as *Babesia* spp. and *Theileria* spp. (Najm et al., 2014). In addition, the marked tendency of foxes to establish populations in suburban and urban areas should be kept in mind, as urbanization is a driving force for the emergence of zoonotic diseases across species and a major risk factor for the transmission of such agents to humans (Hassell et al., 2017). A significant example for dispersal of a fox derived zoonosis is alveolar echinococcosis caused by *Echinococcus multilocularis,* which displays transmission routes similar to HEV including fecal shedding and subsequent ingestion of the pathogen (Vuitton et al., 2015).

In principle, members of the species *Orthohepevirus* C may have zoonotic potential as illustrated by a rat HEV isolate that induced a persistent infection in a human patient (Sridhar et al., 2018). The reservoir for rat associated HEV are invasive *Rattus* species like *R. norvegicus* and *R. rattus* (Ryll et al., 2018) that – analogous to red foxes – globally expand to new (sub-) urban areas and thereby provide the appropriate environment for transmission of wild life associated HEV strains to the human population.

More studies are needed to isolate fox HEV from infected animals, to provide further sequence information about fox-associated HEV and to reveal exposure and infection routes between individuals. This will help to gain a deeper understanding of HEV infection patterns and emergence scenarios at the wildlife-livestock-humans interface.

## Data Availability Statement

The datasets generated for this study can be found in the GenBank BankIt submission, accession number: MN563782.

## Ethics Statement

Ethical review and approval were not required for the animal study because samples were collected from hunted animals during an Echinococcus multilocularis surveillance program conducted in the German federal state of Brandenburg.

## Author Contributions

ME and MG designed the research. ME, LD, and SS performed the experiments. RS, AV-R, and FC analyzed the data. FC provided the animal samples. ME and MG wrote the paper. FC revised the manuscript.

### Conflict of Interest

The authors declare that the research was conducted in the absence of any commercial or financial relationships that could be construed as a potential conflict of interest.

## Supplementary Material

SUPPLEMENTARY TABLE S1 Prevalence of antibodies to Hepatitis E virus among red foxes in the federal state of Brandenburg, Germany. Table compiling total number of tested fox samples. Prevalence estimates per year and the respective two-sided 95% confidence intervals are shown.

SUPPLEMENTARY FIGURE S1 Number of hunted foxes in the federal state of Brandenburg. Source: hunting report Ministry of Rural Development, Environment and Agriculture (https://mlul.brandenburg.de/mlul/de/), German hunting organization (www.jagdverband.de).

SUPPLEMENTARY FIGURE S2 Strip immunoassay recomLine HEV IgG/IgM. ELISA negative fox (1), positive fox (2), positive fox (3), samples. O2N N-terminal part of ORF-2 protein (HEV capsid protein, genotype 1 and 3) O2M central part of ORF-2 protein (HEV capsid protein, genotype 1). O2C C-terminal part of ORF-2 protein (HEV capsid protein, genotypes 1 and 3).

SUPPLEMENTARY FIGURE S3 Western blot analysis of ELISA positive (lanes 1–4) and ELISA negative (lanes 5–9) fox samples.

SUPPLEMENTARY FIGURE S4 Prevalence of antibodies to HEV in fox samples from the districts of Ostprignitz-Ruppin and Prignitz. Prevalence estimates and the respective two-sided 95% confidence intervals are shown. Differences in prevalences were compared by the Fisher exact test und significant differences (*p* < 0.05) indicated by asterics. No samples were received for this region 1995, 2007, and 2008.

SUPPLEMENTARY FIGURE S5 Amino acid alignment of Fox HEV sequences. Fox HEV Germany (#MN563782), fox HEV, the Netherlands (#KC692370), common vole, Hungary (#MH581170), kestrel Hungary (#KU670940), and common vole Germany (#MK192413).

SUPPLEMENTARY FIGURE S6 Development of the hunting index of population density (HIPD) of Brandenburg for the period 1993–2012.
