# Supplementary material for: Spatial-Temporal Dynamics of Hepatitis E Virus Infection in Foxes (Vulpes vulpes) in Federal State of Brandenburg, Germany, 1993–2012
Source: Front Microbiol. 2020 Jan 31;11:115. doi: 10.3389/fmicb.2020.00115 (PMC7005575; doi:10.3389/fmicb.2020.00115)
Supplement: SUPPLEMENTARY TABLE S1 — Prevalence of antibodies to Hepatitis E virus among red foxes in the federal state of Brandenburg, Germany. Table compiling total number of tested fox samples. Prevalence estimates per year and the respective two-sided 95% confidence intervals are shown. [file Data_Sheet_1.PDF]

year	1993	1994	1995	1996	1997	1998	1999	2000	2001	2002	2003	2004	2005	2006	2007	2008	2009	2010	2011	2012	total
total	94	33	23	29	36	47	38	50	50	43	33	51	54	27	48	47	49	75	14	39	880
positive	46	27	21	28	35	45	38	48	45	43	19	42	43	24	31	29	24	58	12	31	689
negative	48	6	2	1	1	2	0	2	5	0	14	9	11	3	17	18	25	17	2	8	191
prevalence [%]	48,9	81,8	91,3	96,6	97,2	95,7	100,0	96,0	90,0	100,0	57,6	82,4	79,6	88,9	64,6	61,7	49,0	77,3	85,7	79,5	81,2
95% CI	38,5-59,5	64,5-93	72-98,9	82,2-100	85,4-99,9	85,5-99,4	90,7-100	86,3-99,5	78,2-96,7	91,8-100	39,2-74,5	69,1-91,6	66,5-89,4	70,8-97,6	49,5-77,8	46,3-75,5	34,4-63,7	66,2-86,2	57,2-98,2	63,5-90,7	-

SUPPLEMENTARY TABLE S1

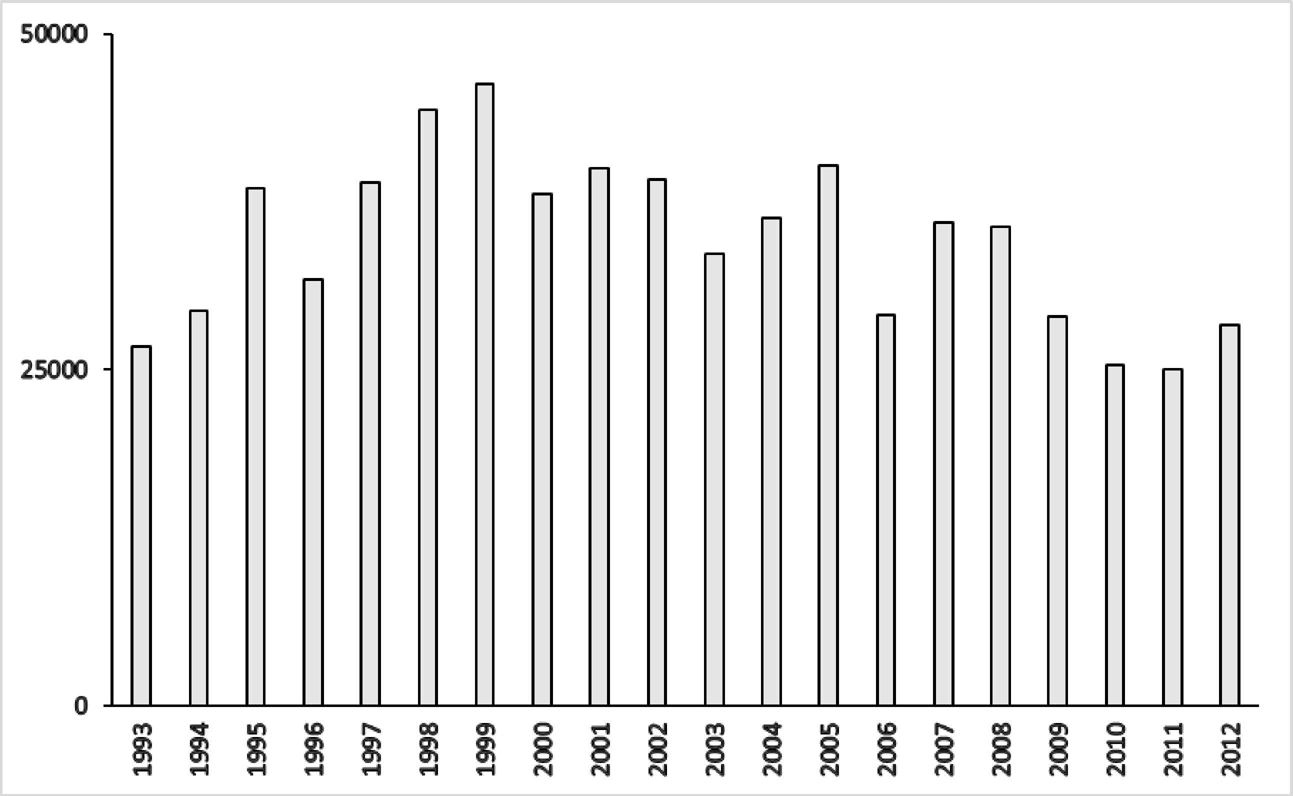

SUPPLEMENTARY FIGURE S1

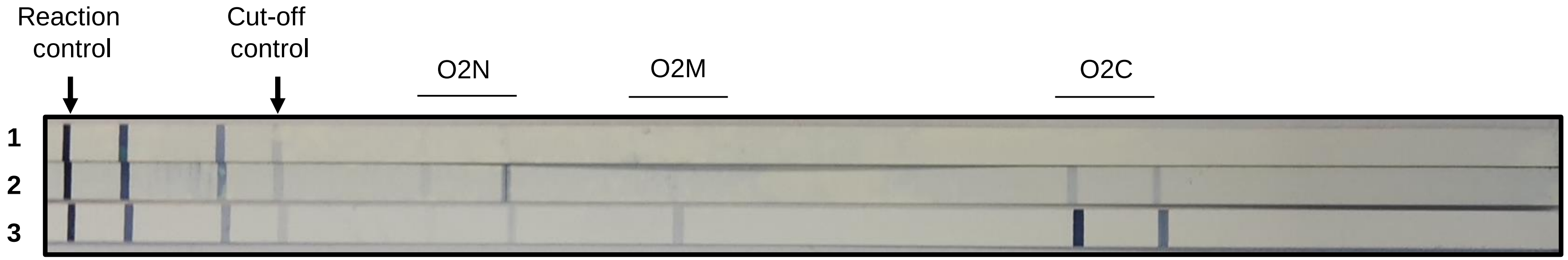

SUPPLEMENTARY FIGURE S2

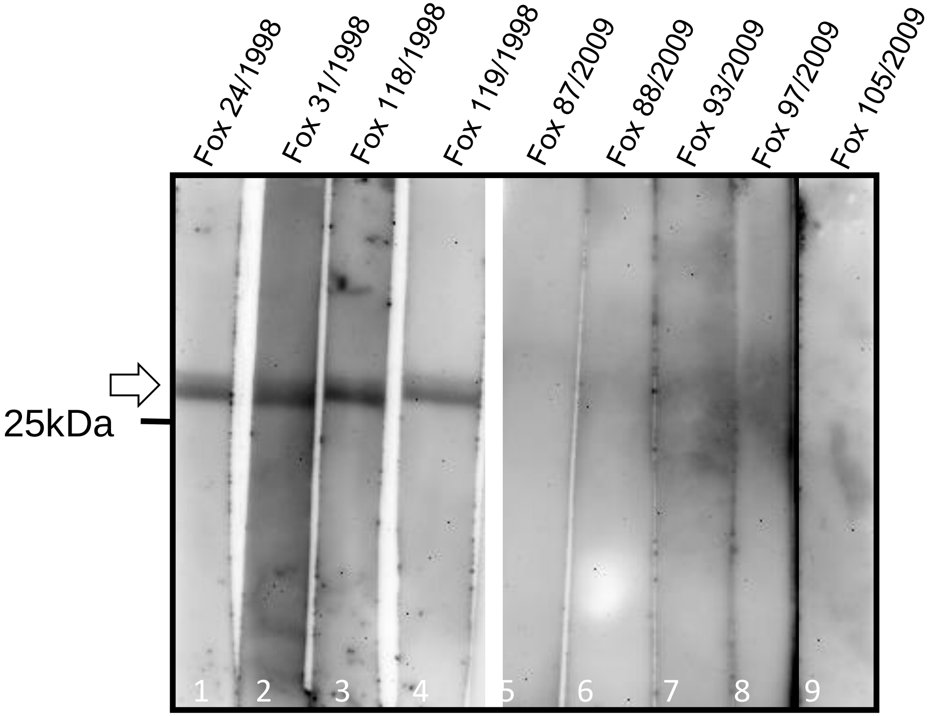

SUPPLEMENTARY FIGURE S3

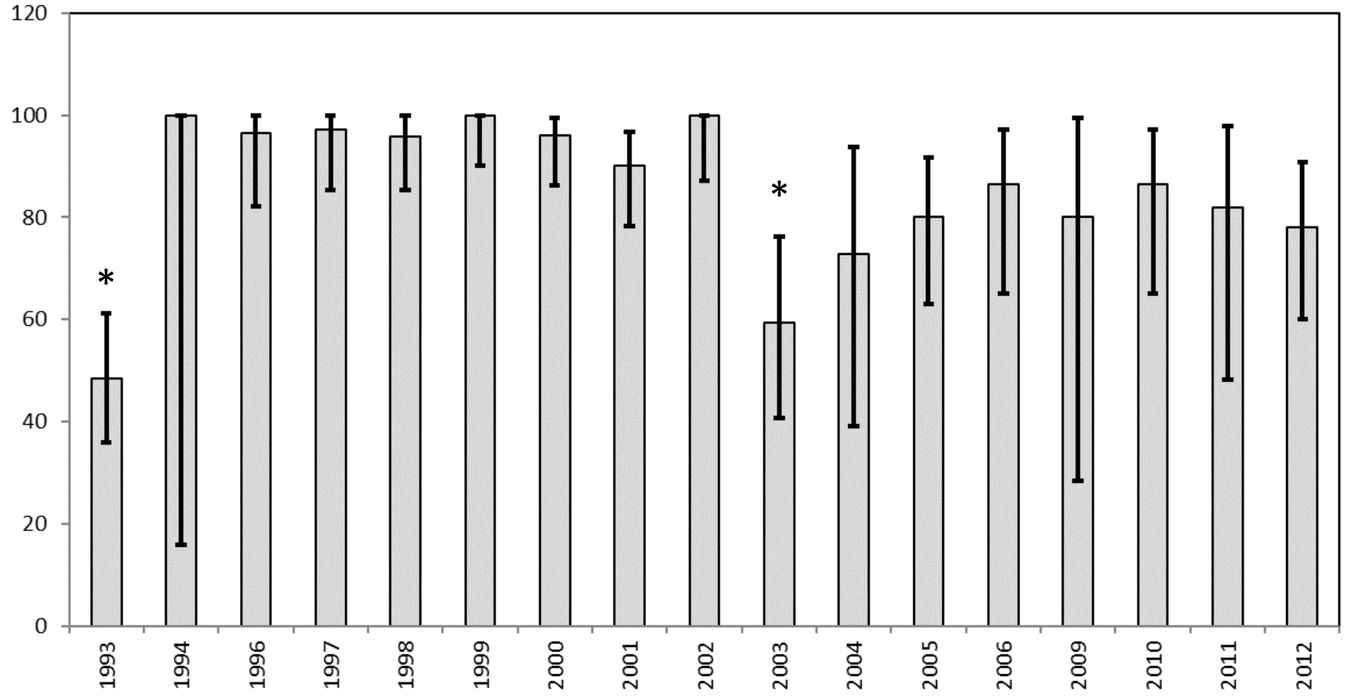

	1993	1994	1996	1997	1998	1999	2000	2001	2002	2003	2004	2005	2006	2009	2010	2011	2012	ges
total	66	2	29	36	47	39	50	50	27	32	11	35	22	5	22	11	32	**516**
positive	32	2	28	35	45	39	48	45	27	19	8	28	19	4	19	9	25	**432**
negative	34	0	1	1	2	0	2	5	0	13	3	7	3	1	3	2	7	**84**
prevalenc	48	100	97	97	96	100	96	90	100	59	73	80	86	80	86	82	78	
95% CI	36-61,1	15,8-100	82,2-99,9	85,4-99,9	85,4-99,8	90-100	86,2-99,5	78,2-96,7	87,2-100	40,6-76,3	39,0-93,8	63,1-91,6	65,1-97,1	28,3-99,5	65,1-97,1	48,2-97,8	60,0-90,7	

SUPPLEMENTARY FIGURE S4

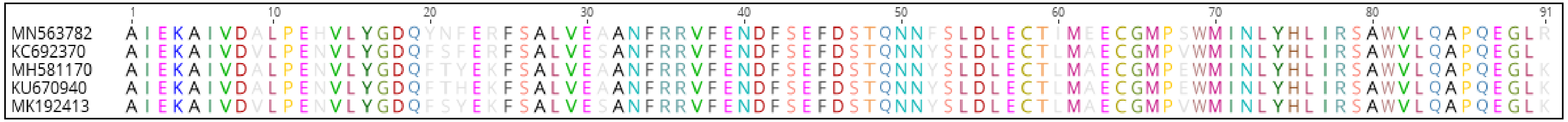

SUPPLEMENTARY FIGURE S5

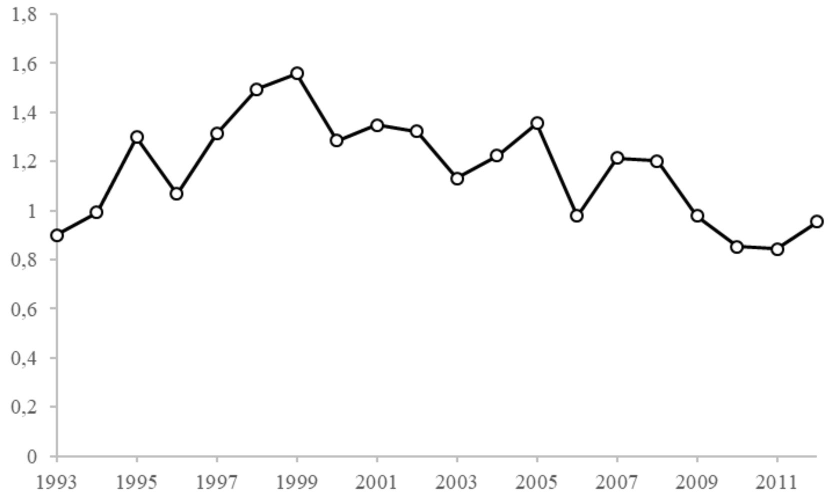

SUPPLEMENTARY FIGURE S6
